# Bioinformatics analysis of the early inflammatory response in a rat thermal injury model

**DOI:** 10.1186/1471-2105-8-10

**Published:** 2007-01-10

**Authors:** Eric Yang, Timothy Maguire, Martin L Yarmush, Francois Berthiaume, Ioannis P Androulakis

**Affiliations:** 1 Biomedical Engineering Department, Rutgers University, Piscataway, NJ, USA; 2 Center for Engineering in Medicine/Surgical Services, Massachusetts General Hospital, Harvard Medical School, and the Shriners Hospitals for Children, Boston, MA, USA

## Abstract

**Background:**

Thermal injury is among the most severe forms of trauma and its effects are both local and systemic. Response to thermal injury includes cellular protection mechanisms, inflammation, hypermetabolism, prolonged catabolism, organ dysfunction and immuno-suppression. It has been hypothesized that gene expression patterns in the liver will change with severe burns, thus reflecting the role the liver plays in the response to burn injury. Characterizing the molecular fingerprint (i.e., expression profile) of the inflammatory response resulting from burns may help elucidate the activated mechanisms and suggest new therapeutic intervention. In this paper we propose a novel integrated framework for analyzing time-series transcriptional data, with emphasis on the burn-induced response within the context of the rat animal model. Our analysis robustly identifies critical expression motifs, indicative of the dynamic evolution of the inflammatory response and we further propose a putative reconstruction of the associated transcription factor activities.

**Results:**

Implementation of our algorithm on data obtained from an animal (rat) burn injury study identified 281 genes corresponding to 4 unique profiles. Enrichment evaluation upon both gene ontologies and transcription factors, verifies the inflammation-specific character of the selections and the rationalization of the burn-induced inflammatory response. Conducting the transcription network reconstruction and analysis, we have identified transcription factors, including AHR, Octamer Binding Proteins, Kruppel-like Factors, and cell cycle regulators as being highly important to an organism's response to burn response. These transcription factors are notable due to their roles in pathways that play a part in the gross physiological response to burn such as changes in the immune response and inflammation.

**Conclusion:**

Our results indicate that our novel selection/classification algorithm has been successful in selecting out genes with play an important role in thermal injury. Additionally, we have demonstrated the value of an integrative approach in identifying possible points of intervention, namely the activation of certain transcription factors that govern the organism's response.

## Background

Thermal injury is among the most severe forms of trauma and its effects are both local and systemic. Response to thermal injury includes cellular protection mechanisms, inflammation, hypermetabolism, prolonged catabolism, organ dysfunction and immuno-suppression [1]. Changes in energy expenditure following burn injury have been attributed to processes such as gluconeogenesis, ureagenesis, fatty acid synthesis and catabolism, processes relating to the need to compensate for the increased loss of body heat through the injured skin, as well as changes in the circulating levels of plasma proteins primarily synthesized in the liver [2]. Therefore, physical stress as a result of burn has a significant impact on the liver, an organ that plays a critical role in modulating immune function, inflammatory processes and the acute phase response in the attempt to restore homeostasis.

It has been hypothesized that gene expression patterns in the liver will change with severe burns, thus reflecting the role the liver plays in the response to burn injury. Characterizing the molecular fingerprint (i.e., expression profile) of the inflammatory response resulting from burns may help elucidate the activated mechanisms and suggest new therapeutic intervention. To record the transcriptional characteristics of hypermetabolism following severe injury, various animal models have been proposed to quantify *in vivo *the appropriate gene expression response [3-6]. Transcriptional profiling used in the context of monitoring burn-induced inflammatory responses [1,4,7,8] may eventually provide the detailed mechanism behind burn injury if information can be extracted from the reams of data generated. It is our belief that relevant genes tend to be part of large highly correlated clusters due to the coordinated actions of these genes and can therefore be isolated if one were to utilize clustering to obtain sets of highly correlated gene and combine it with a selection step that denotes clusters as relevant based upon their population.

Even though individual components of the overall inflammatory response have received intense scrutiny, deciphering the cross-talk between components is a daunting task due to the extraordinary complexity of the inflammatory response, thus necessitating an integrative approach [9] that requires the combination of outside information besides strictly gene expression levels or metabolic flux levels. While gene expression in inflammation is not solely transcriptionally controlled [10] the first step towards understanding inflammation is to evaluate possible mechanisms which give rise to expression data, the most readily available source of cellular response.

In this paper we propose an integrated framework for analyzing time-series transcriptional data, with emphasis on the burn-induced response within the context of the rat animal model. The proposed approach is composed of three elements:

1. Novel characterization of the dynamic transcriptional response

2. Identification of maximally informative genes

3. Elucidation and quantification of regulatory interactions

Our analysis robustly identifies critical expression motifs, indicative of the dynamic evolution of the inflammatory response, and subsets of informative genes and their associated metabolic pathways, thus integrating extracted genes with known networks of interaction. We will demonstrate how, based on the reduced set of informative genes that are optimally selected, we can construct a potential network of regulatory interactions and suggest potential targets for further investigation and intervention.

## Results

Following severe trauma, the liver plays a crucial role in mediating a host of physiological responses. These processes include an increase in energy expenditure [4], the production of acute phase proteins [11], activation of the complement, kinin, clotting, and fibrinolytic pathways [12-14], the initiation of immune response to prevent against later onset of sepsis, as well as the up-regulation of mechanisms to prevent against oxidative damage induced by the activation of these responses [15]. Through our robust analyses we have identified 4 motifs which capture many of these underlying biological mechanisms, as well as the expected temporal responses.

To dissect the onset of inflammation, we have summarized the key physiological components, as identified through ontology searches, listed in Table 1, We have further subdivided these components into 2 major groups: 1) those processes which fit within the global characterization of metabolism, as a means to verify our approach, since a large body of work has been established to characterize these responses; 2) other processes which we have detected that are integral in the inflammation process, but have not been documented in detail in the literature. We have also subdivided the inflammation process into three phases: 1) early (up to four hours); 2) middle (up to 8 hours); and 3) late (following 8 hours up to the 24 hour time point).

**Table 1:** Gene Ontology Enrichment of Informative motifs

**Process Name**	**Motif 1**	**Motif 2**	**Motif 3**	**Motif 4**
protein biosynthesis	**0.000**	0.082	0.568	1.000
ribosome biogenesis	**0.000**	0.314	1.000	1.000
response to unfolded protein	**0.003**	0.268	1.000	1.000
protein folding	**0.004**	0.178	1.000	1.000
peptidyl-arginine methylation, to asymmetrical-dimethyl arginine	**0.015**	1.000	1.000	1.000
protein-nucleus export	**0.023**	1.000	1.000	1.000
adenine metabolism	**0.023**	1.000	1.000	1.000
response to stress	**0.023**	1.000	1.000	1.000
pyridoxine biosynthesis	**0.030**	1.000	1.000	1.000
endothelial cell differentiation	**0.030**	1.000	1.000	1.000
hormone-mediated signaling	**0.038**	1.000	1.000	1.000
re-entry into mitotic cell cycle	**0.045**	1.000	1.000	1.000
protein amino acid prenylation	**0.045**	1.000	1.000	1.000
transmission of nerve impulse	**0.045**	1.000	1.000	1.000

negative regulation of calcium-mediated signaling	1.000	**0.000**	1.000	1.000
Acute phase response genes	1.000	**0.000**	1.000	1.000
ubiquitin-dependent protein catabolism	0.288	**0.001**	0.284	1.000
ureteric bud development	1.000	**0.005**	1.000	1.000
nucleosome assembly	1.000	**0.013**	0.128	1.000
protein catabolism	1.000	**0.015**	1.000	1.000
homophilic cell adhesion	0.149	**0.017**	1.000	0.143
norepinephrine biosynthesis	1.000	**0.018**	1.000	1.000
protein refolding	1.000	**0.027**	1.000	1.000
chaperone cofactor dependent protein folding	1.000	**0.027**	1.000	1.000
N-acetylglucosamine metabolism	1.000	**0.036**	1.000	1.000
thyroid hormone catabolism	1.000	**0.036**	1.000	1.000

cellular response to starvation	1.000	1.000	**0.000**	1.000
negative regulation of Ras protein signal transduction	1.000	1.000	**0.000**	1.000
RNA processing	1.000	1.000	**0.001**	1.000
cell glucose homeostasis	1.000	1.000	**0.002**	1.000
protein amino acid dephosphorylation	0.436	0.494	**0.003**	0.421
cytokinesis	1.000	1.000	**0.003**	1.000
nucleocytoplasmic transport	1.000	1.000	**0.005**	1.000
negative regulation of transcription, DNA-dependent	0.116	1.000	**0.007**	1.000
somitogenesis	1.000	1.000	**0.015**	1.000
glycogen metabolism	1.000	1.000	**0.015**	1.000
thioredoxin pathway	1.000	1.000	**0.023**	1.000
negative regulation of Wnt receptor signaling pathway	1.000	1.000	**0.023**	
negative regulation of neuron differentiation	1.000	1.000	**0.023**	1.000
frizzled signaling pathway	1.000	1.000	**0.030**	1.000
tRNA processing	1.000	1.000	**0.030**	1.000
regulation of Wnt receptor signaling pathway	1.000	1.000	**0.030**	1.000
interleukin-2 biosynthesis	1.000	1.000	**0.030**	1.000
RNA-nucleus export	1.000	1.000	**0.037**	1.000
Golgi organization and biogenesis	1.000	1.000	**0.037**	1.000
regulation of transcription, DNA-dependent	0.077	0.166	**0.040**	1.000

grooming behavior	1.000	1.000	1.000	**0.015**
medium-chain fatty acid transport	1.000	1.000	1.000	**0.022**
embryonic placenta development	1.000	1.000	1.000	**0.022**
catecholamine catabolism	1.000	1.000	1.000	**0.022**
inflammatory response	0.494	1.000	1.000	**0.028**
inflammatory response	0.494	1.000	1.000	**0.028**
embryo implantation	1.000	1.000	1.000	**0.029**
synaptic vesicle endocytosis	1.000	1.000	1.000	**0.029**
response to acid	1.000	1.000	1.000	**0.036**
associative learning	1.000	1.000	1.000	**0.036**
nitrogen fixation	1.000	1.000	1.000	**0.036**
regulation of dopamine metabolism	1.000	1.000	1.000	**0.036**
mesoderm cell differentiation	1.000	1.000	1.000	**0.036**
regulation of transcription	1.000	1.000	1.000	**0.038**
vasculogenesis	1.000	1.000	1.000	**0.043**
fatty acid transport	1.000	1.000	1.000	**0.043**

In the early phases of inflammation, we see a majority of those processes which can be grouped as metabolic in nature exhibiting distinct temporal changes. For example, motif 4, which displays a peak in up-regulation within the first 2 hours following burn injury, contains genes which are primarily responsible for the transport of fatty acids and triglycerides into the cell. Cluster 3, characterized by genes involved in FA oxidation clearly demonstrates an early up-regulation followed by significant down-regulation. Cluster 3 is also actively involved in fatty acid transport. Furthermore, the CCAAT/Enhancer Binding Protein a known transcription factor for gluconeogenesis [16] is a key regulator of cluster 4. These coupled processes of fatty acid transport and breakdown have been shown to be activated quite early in the inflammatory response, and it has been hypothesized that they allow the liver to build up energy stores in the form of ATP for the later production of acute phase proteins [4,17]. One final ontology, related to cellular energetics and the derivation of energy stores, is glycogen metabolism, is also present in motif 3. Taken together, one may conclude that the utilization of fatty acids, and other energy sources, happens very early on following thermal injury, a point which is validated through biochemical analysis of free fatty acid levels in burn injury in vivo models [4].

Within the initial temporal phases of inflammation, our approach has identified biological processes above and beyond those categorized as cellular energetics. For example, we have identified ontologies involved in catecholamine metabolism and inflammation. Endogenous catecholamines are primary mediators of the hypermetabolic response to burn [18,19]. Shortly after severe burn, plasma catecholamine levels have been observed to increase significantly. Consistent with this observation, cluster 4 is enriched in catecholamine metabolism genes. The inflammatory process within this time period is defined in large part by the initiation of the complement and kinin and cascade systems, present in motif 4. Basically, two genes, murinoglobulin 1 homolog (alpha 1-inhibitor 3) and complement component 5 receptor 1 (C5AR1) regulate these key inflammatory/acute phase responses in an attempt to dampen the overall inflammatory response so as to prevent it from progressing to a chronic state [20,21]. During the middle temporal phase we have identified genes involved in the acute phase response, ubiquitin dependent protein catabolism, and interleukin 2 (Il-2) synthesis. Cluster 2 is enriched with genes associated with the acute phase response and also exhibits the most significant enrichment in the known inflammatory Transcription Factors (TF) NF-kβ and HNF1. Ubiquitin catabolism is a major mechanism of muscle wasting characteristic of hypermetabolic states and systemic inflammation [22]. Up-regulation of associated genes initiated in this middle temporal phase and is pronounced at latter stages, as indicated by the ontology enrichment of Cluster 2. Interleukin 2 and its receptor have also been discovered to mediate the acute phase response and dysfunction within the liver [23]. Known to regulate the production and activity of many inflammatory mediators and cells, Interferon Regulatory Factors (IRF) [24] were identified as a key transcription factor family of Cluster 3 which is primarily responsible for interleukin-2 biosynthesis.

In the final temporal portion of the acute phase response to thermal injury, we have identified processes which can be grouped into two major components which again, are unique to our analysis procedure: 1) RAS-RAC signaling cascade (motif 3); 2) response to stress (motif 1). Within motif 3, which has a secondary peak at 24 hours, we have identified the gene for protein phosphatase 2a, catalytic subunit, beta (Ppp2cb) which is a negative regulator of RAS-RAC signaling, which in turn will down-regulated RAS induced activation of NF-kB pathways [25], and will provide a late-stage mechanism and like the effect of alpha 1-inhibitor 3 and C5AR1 prevent a transition to a chronic inflammatory state. Late stage stress response is also a beneficial process and is aimed at attenuating the stress response. For example we have identified glycogen synthase kinase 3 beta (GSK-3 beta) within motif 1 which exhibits a 24 hour peak. GSK-3 beta is known to be a key element in the switch from acute to chronic/systemic inflammatory response [26]. Another interesting finding in this late stage inflammatory response is the up-regulation of two pathways, involved in generating large pools of thioredoxin and N-acetylglucosamine. In [27] the levels of thioredoxin were measured in severely burned patients, and noticeable increases were observed, interestingly characterized by two peaks of increase. Cluster 2 is enriched in genes involved in this particular pathway. It was also recently observed [28] that the acute-phase response is accompanied by increased liver pools of N-acetylglucosamine at about 12 h post inflammation. Consistent with this observation, Cluster 1 is enriched in genes of that ontology. Interestingly glucosamine is currently considered as a dietary supplement for wound healing [29].

In addition to the genes which are active in the aforementioned responses we have also assembled the set of transcription factors for all the genes involved in the four maximally informative motifs by making use of Trafac [30], which runs the Genomatix MatInspector analysis suite in the background. We ran two sets of analysis one upon the transcription factors which were enriched at a statistically significant level (Table 2), and those that showed large deviations after the Network Component Analysis (NCA) operation.

**Table 2:** Transcription factor enrichment of informative motifs

**Transcription Factor**	**Motif 1**	**Motif 2**	**Motif 3**	**Motif 4**
AHR-arnt heterodimers and AHR-related factors	**0.00**	0.37	0.45	0.36
E-box binding factor without transcript. activation	**0.03**	0.74	0.78	0.78
Brn POU domain factors	**0.03**	0.19	0.19	0.23
CAS interating zinc finger protein	**0.03**	0.22	0.28	0.15
MYOblast Determining factor	**0.04**	0.13	0.23	0.16
GC-Box factors_SP1/GC	**0.05**	0.15	0.24	0.12
Cell cycle regulators: Cell cycle dependent element	**0.05**	0.64	0.69	0.70
Promoter CCAAT binding factors	**0.08**	0.20	0.16	0.31
RBPJ – kappa	**0.09**	0.27	0.28	0.17

C-myb, cellular transcriptional activator	0.28	**0.00**	0.28	0.15
CP2-erythrocyte Factor related to drosophila Elf1	0.24	**0.00**	0.26	0.36
Homeodomain factor aberrantly expressed in myeloid leukemia	0.23	**0.02**	0.23	0.23
OCT1 binding factor (POU-specific domain)	0.25	**0.06**	0.25	0.14
AP4 and Related proteins	0.11	**0.09**	0.18	0.23
MAF and AP1 related factors	0.27	**0.09**	0.27	0.23

NKX/DLX – homeodomain sites	0.96	0.74	**0.00**	0.20
Interferon Regulatory Factors	0.18	0.91	**0.00**	0.18
CLOX and CLOX homology (CDP) factors	0.25	0.73	**0.00**	0.52
p53 tumor suppr.-neg. regulat. of the tumor suppr. Rb	0.12	0.21	**0.00**	0.28
Basic and erythroid Krueppel like factors	0.17	0.24	**0.01**	0.17
Pancreatic and intestinal homeodomain transcr. factor	0.23	0.20	**0.02**	0.24
Microphthalmia transcription factor	0.37	0.24	**0.02**	0.37
Human and murine ETS1 factors	0.95	0.86	**0.07**	0.36

Regulator of B-Cell IgH transcription	0.28	0.22	0.28	**0.03**
Hypoxia inducible factor, bHLH/PAS protein family	0.37	0.36	0.43	**0.04**
E-box related factors	0.37	0.36	0.37	**0.07**
ZF5 POZ domain zinc finger	0.31	0.11	0.60	**0.08**
PAX-2 binding sites	0.37	0.24	0.22	**0.08**
CCAAT/Enhancer Binding Protein	0.16	0.19	0.23	**0.09**

E2F-myc activator/cell cycle regulator	**0.02**	**0.04**	0.27	0.20
Vertebrate caudal related homeodomain protein	**0.04**	**0.05**	0.23	0.23
FAST-1 SMAD interacting proteins	**0.08**	**0.05**	0.13	0.12
AMV-viral myb oncogene	**0.10**	**0.04**	0.11	0.20

Camp-Responsive Element Binding proteins	**0.08**	0.17	**0.08**	0.12

Octamer binding protein	1.00	**0.00**	**0.03**	0.96
AARE binding factors	0.31	**0.04**	**0.08**	0.37
Nuclear Factor Kappa B/c-rel	0.22	**0.05**	**0.08**	0.22

Zinc binding protein factor	0.20	**0.01**	0.21	**0.09**
Hepatic Nuclear Factor 1	0.15	**0.04**	0.23	**0.07**

Of special interest are hypoxia inducible factor, p53 tumor suppressor (P53), and Cas-interacting zinc finger protein. Severe burns typically cause a hypovolemic shock response during the first 24 hours; therefore, it is plausible that there was reduced oxygen delivery to the liver resulting in hypoxia. One way that cells respond to hypoxia is through increased activation of the hypoxia inducible factor (HIF), which is thought to enhance cellular adaptation to low oxygen. Recent evidence verified the stimulation of HIF by well known inflammatory signals, such as Tumor Necrosis Factor (TNF) and Interleukin 1 (IL-1), which results in the transcription of several genes leading to proteins that increase blood flow [31]. Cluster 3 is enriched in hypoxia-related genes and, furthermore, HIF is a leading TF for the genes within that cluster based on the corresponding TF-enrichment analysis. p53 has been reported elevated during inflammation in several studies. Specifically, p53 represses MAPK as well as RAS signaling pathways [32], both of which play a major role in signaling of the inflammatory response [33]. Thus, p53 may be an important factor for the down-regulation of the acute inflammatory response. Aside from hypoxia as an outcome of inflammation, it has been shown [34] that thermal injury exhibits an interplay between liver cell apoptosis and proliferation while attempting to establish a trend towards homeostasis. Among the regulators associated with cluster 1 we identified Cas-interacting zinc finger protein (CIZ) which is a known regulator of the bone morphogenetic protein (BMP) signal regulating apoptosis [35]. Furthermore, Aryl Hydrocarbon Receptor (AhR) is a ligand-activated transcription factor known to influence apoptosis, conceivably by regulating the expression of genes involved in apoptotic signaling [36].

Taken together, these three parallel approaches (motif identification, ontology enrichment, transcription factor quantification) allow us to identify multiple layers of the inflammatory response process to thermal injury. It should be noted that all three approaches are needed in combination, being that the control elements we have identified as transcription factors are not contained within the four motifs. This phenomenon may be explained by one or both of the following. First, the motif identification algorithm itself has been established to identify motifs that contain a large quantity of genes, and the regulatory elements we have found are contained in motifs with lower quantities of genes. Second, these regulatory elements exhibit different temporal profiles then those of the four motifs, since they work on a different time scale. Thus, these regulatory elements, which exist higher in the signaling cascade, may be immediately up-regulated in the inflammatory process, and demonstrate their delayed effect in the up-regulation and down-regulation of the large clusters of genes present in each of the motifs.

## Discussion

**Figure 1:**
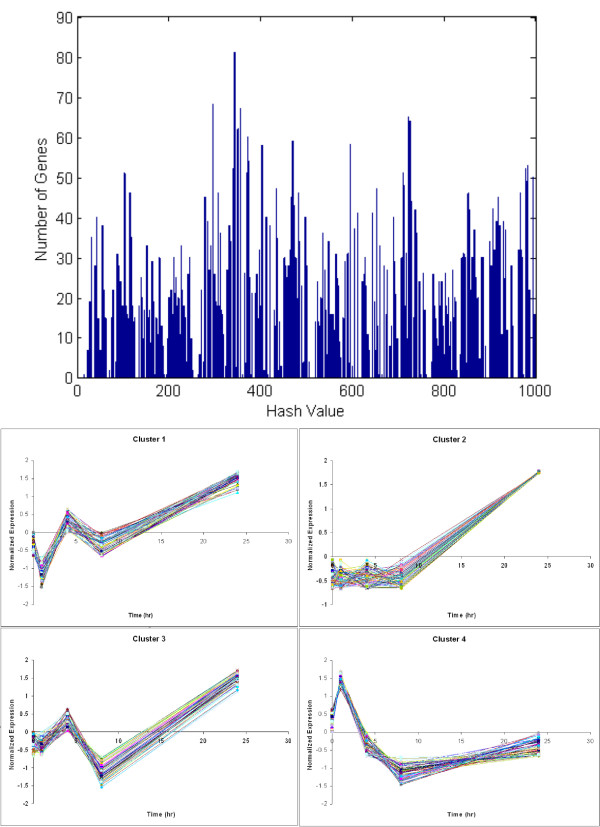
Motif Distribution(top) and expression profile of the selected genes(bottom). Cluster 1–4 have been selected by the algorithm as being informative.

The symbolic transformation of the gene expression profiles, followed by the proposed hashing, results in a fine-grained clustering of the expression profiles, as shown in the top part of Figure 1. Each peak indicates the number of transformed expression profiles that hash to a particular motif value. All such expression profiles will have identical symbolic representations and as a result, very similar raw expression profiles. The z-score transformation eliminates differences due to magnitude, thus the intensity of the signal is not taken explicitly into account. However, at this point we will assume that if two genes have similar normalized profiles they should both be considered for further analysis regardless of the differences in magnitude. With each peak there is an associated transformed average profile and typical examples are depicted in the lower part of Figure 1. Thus, the combination of the symbolic representation and hashing allowed the identification of a large number of potential clusters of genes whose transformed expression profiles are identical. We term those "expression motifs." It is important to realize that similarity is based now on the fact that similar motifs hash to the same value and not to some point-wise distance metric (Euclidean or other). As seen in Figure 1, each of the motifs contains expression profiles which are highly correlated and tightly grouped; pointing to the overall quality of the hash based clustering in terms of intra-cluster variance, supporting our use of a hashing based methodology in creating the initial clusters.

**Figure 2:**
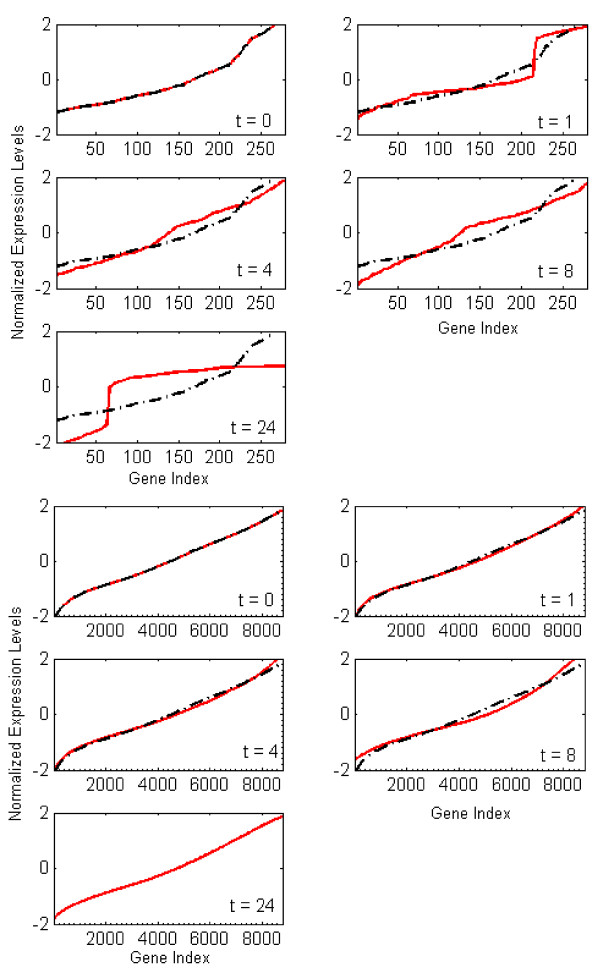
The evolution of the transcriptional state vs. time. (Top) The transcriptional state of an informative set of genes. (Bottom) The transcriptional state of the entire array.

The fine-grained clustering provides a potential, albeit enormous, number of tentative clusters. However, our assumption is that the underlying dynamic response of the transcriptional experiment can be expressed in terms of a smaller number of expression "motifs". When performing our selection step, we selected 4 motifs containing a total of 281 gene probes. The transcriptional state which corresponds to the most informative genes also illustrates an interesting dynamic insomuch that a two wave burn response as observed by [37] is evident, as shown in Figure 2. What we can see is that at hours 1 and 24, time points previously identified as critical points in the evolution of the burn response, a distinct breakpoint between the low and high expression levels for the informative genes is evident; something which is not seen at the time points 4 and 8 hours. This is in contrast to a transcriptional state which includes all of the genes. By including all of the genes the dynamics are not visible, especially the two events which have been previously observed. Given the clear evidence of two critical events in our informative set of genes, we believe that it is reasonable to state that we have selected genes which are playing a critical role in the short-term evolution (i.e. the first 24 hours) of the burn response.

**Figure 3:**
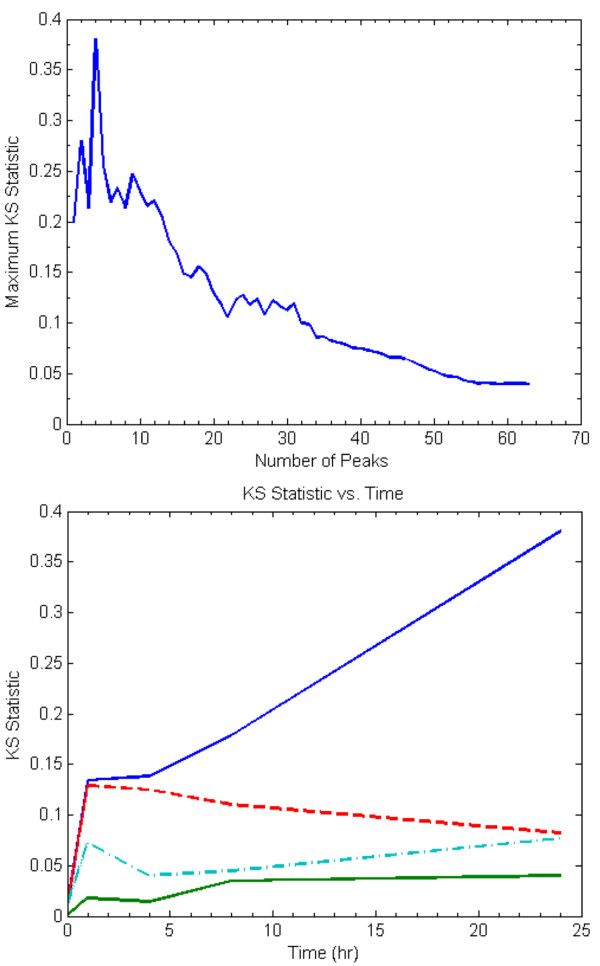
KS-metric evolution vs. number of peaks added (top). KS-metric temporal evolution of informative vs. uninformative genes (bottom).

These probes were selected due to their ability to exhibit the greatest change within their associated transcription state. In Figure 3a we can see that the addition of a single peak, the Kolmogorov-Smirnov (KS) statistic has an intermediate value, and as more peaks are added, it reaches a maximum at four, after which it decreases. The presence of this maximum allows us to assert that our algorithm has managed to extract a set of genes in which the changes in an organism's response is most evident.

### Randomized Testing

In both of the randomized testing cases Figure 3b, it is evident that neither the case where motifs were randomly selected nor the case in which genes were randomly selected did the KS Statistic show as great a deviation as found under the greedy selection heuristic. While this does not preclude the existence of a better globally optimal solution, it does however suggest that our current heuristic is a reasonable approach to finding a set of optimal of motifs that reflects the underlying dynamics of the system.

### Identification of significant processes and regulators

**Figure 4:**
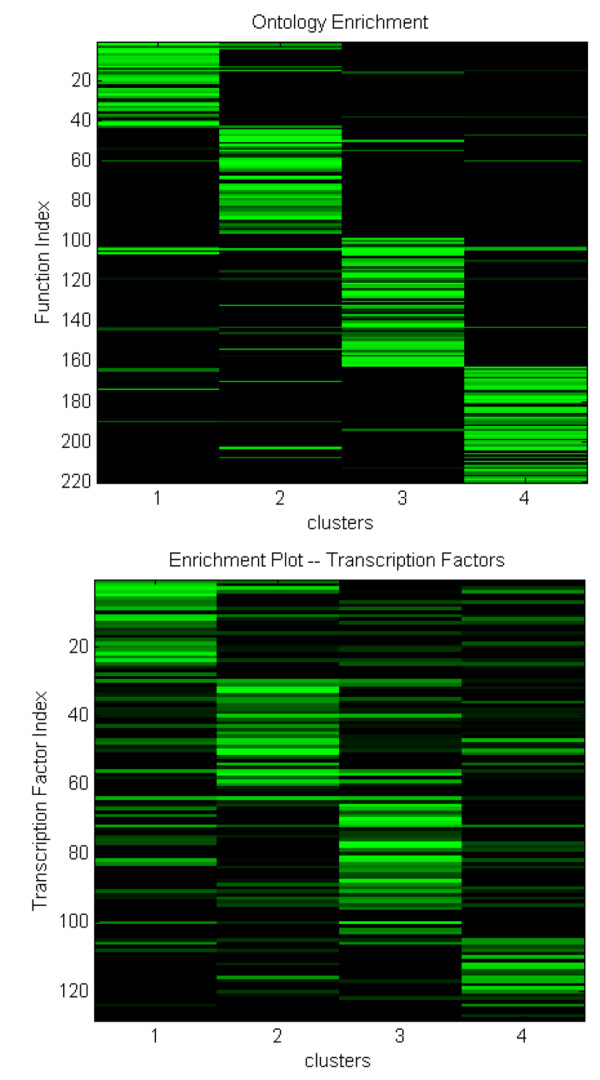
Relative probability of a particular transcription factor binding to any given cluster. The transcription factor index is an ID number specifying each transcription factor numbered 1-N, where N is the number of transcription factors in our analysis. The brighter the color, the more statistically significant the transcription factor enrichment.

Figure 4 shows the localization of the ontology and the transcription factors. What can be clearly seen here is the diagonally dominant aspect of both the gene ontologies as well as the transcription factors. This supports the initial contention by [38], which states that correlated genes exhibit similar functions and regulatory mechanisms. It also verifies the applicability for the utilization of hashing to conduct the initial clustering. More importantly, we believe that such a result validates the shape based approach implemented since significant processes and regulators have been selected by considering shape alone.

By selecting ontologies and transcription factors that are enriched with (P < .05), we have identified hypothetical processes and regulators behind thermally induced inflammation. Genes involved in the acute phase response, inflammation, fatty acid metabolism, cholesterol import (Table 1) were found to be significantly enriched within our cluster, all with a p-value less than .05, and the acute phase response showing a p-value on the order of 1 × 10^-5^. The significance of these ontologies is that in addition to being statistically significant in our selected genes, they are also known to be significant processes that occur during severe thermal injury. From this ontology result, we believe that our algorithm has shown the ability to extract genes which are involved with the overall biological response to burn.

**Figure 5:**
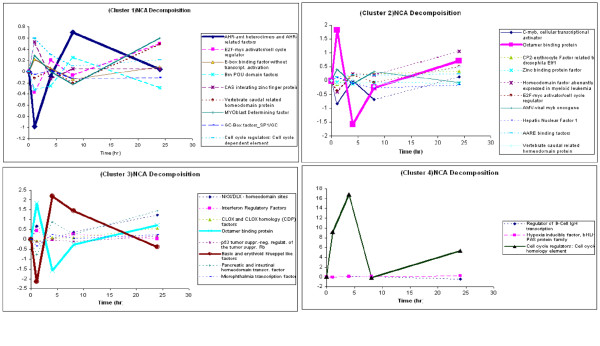
Typical profiles of Transcription Factor Activity Obtained from NCA. The transcription factors in bold are hypothesized as being more important based upon the scale of their activity. The cutoff was calculated by taking the transcription factor that showed the greatest difference over the experimental time period. Other transcription factors were included if their maximum difference was within the bootstrapped confidence intervals [85] of the originally selected profile.

Given the set of statistically enriched transcription factors given in Table 2, the relative dynamics of the transcription factors predicted via NCA for the genes associated with these transcription factors are given in Figure 5. From these plots, it is evident that the majority of the transcription factors show activity within a narrow range of expression levels, while a relatively few transcription factors show expression levels which differ greatly over the experimental time course. It is our hypothesis that these highly active transcription factors represent important parts of the signaling process. The identification of transcription factors allows us to precisely target unwanted responses through techniques such as siRNA without disrupting the overall signaling cascade.

### Identification of hypothetical primary regulators

What we can extract from Figure 5, is a set of transcription factors which can be hypothesized to be important in the response of each cluster of genes to the initial burn injury. The transcription factors that NCA identified as highly active are the aryl hydrocarbon receptors, octamer binding units, erythroid kruppel like factors, and cell cycle homology elements (Table 2). The presence of the octamer binding units can be rationalized due to the generalized stress response[39] of the organism leading to the initiation of the immune response normally observed during burn[40], while the presence of the aryl hydrocarbon receptor and cell cycle homology elements can be rationalized by the parts that they play in the cell cycle and cell regeneration[41]. Finally the presence of the erythroid kruppel factors coupled with its role as a pro-inflammatory initiator[42] suggests a possible role for it in the inflammatory response associated with burn injury.

### Analysis of gene interaction networks

**Figure 6:**
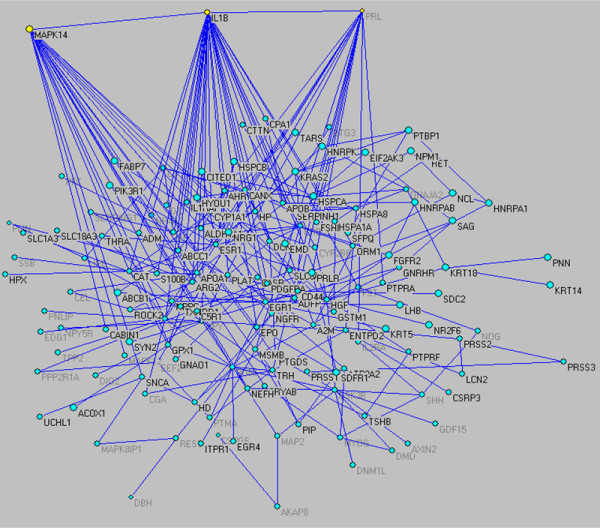
Gene interaction network formed by the informative genes associated with the burn-induced inflammatory response. For better visual inspection the highly interconnected hubs has been isolated and clearly indicated.

Metabolic networks are known to exhibit small-word characteristics [43] with average path length significantly smaller than the corresponding length of a random network. The small word properties in addition to the existence of hubs give gene interaction networks some of their key distinct characteristics, namely: (i) local perturbations are quickly propagate across the entire network as nodes interact with each other via the hub proteins; (ii) the existence of hubs proteins is advantageous because it identifies key controls whose manipulation can have significant effects such as controlling the onset of a detrimental process and thus identify major points of intervention; and (iii) hubs make these networks prone to quick deterioration should one of the key controls be attacked [44]. Therefore, hubs proteins play a critical, important, role thus requiring additional attention. Through our analysis we have determined three major hubs of activity, within our protein interaction network, those being interleukin1-beta (Il-1B), prolactin (PRL), and mitogen activated protein kinase 14 (MAPK14; p38 MAPK). Il-1B has been reported to be a dominant cytokine that acts as a central regulator of the acute inflammatory response, basically through the production of acute phase proteins [45]. This is evident in the large cascade of genes influenced through the activities of Il-1B (Figure 6). In addition, one specific cascade which is initiated through the activity of Il-1B, is that regulated by PRL, another of the dominant nodes we identified [46]. While Il-1B has the outcome of up-regulating a variety of genes needed in mediating the acute phase response, PRL has the inverse effect, in that it aides in the acute phase response by opposing the immunosuppressive effects of glucocorticoids and other inflammatory mediators to maintain steady-state homeostasis [47,48]. The third hub we identified, p38MAPK, has also been established as a prominent gene involved in the acute phase response [49-52]. The p38 signaling cascade exhibits its effects following thermal injury, generally through the up-regulation of proinflammatory cytokines, such as the aforementioned Il-1B [53]. Thus, not only are these hubs capable of regulating a variety of down-stream genes, they themselves exhibit a high-degree of cross-talk, and regulate each other within the overall context of the protein interaction network. In addition, identification of these hubs provides potential therapeutic targets, to mitigate the inflammatory response observed following thermal injury.

### Comparisons with other clustering algorithms

Finally, we comparatively evaluated several clustering algorithms, all of which are publicly available, such as STEM (Short Time-series Expression Miner) [54], hierarchical clustering [55] and k-means based on the Matlab Bioinformatics Toolbox to determine the relative enrichment of ontologies and transcription factors [56,57]. With the exception of STEM which has a built-in selection criterion based on the frequency of an expression pattern, the other methods do not perform a selection but rather cluster all responses. The results of Table 3 demonstrate the advantages of the proposed analysis in terms of the ability to enrich both ontologies and TFs in relevant processes and transcription factors. It is important to realize that even though STEM appears to enrich the clusters more than our motifs method, our approach takes into account the dynamic responses that actually affect the experiment therefore it achieves significantly superior enrichment in terms of inflammation-specific ontologies and TFs. Hence the comparative results provide a strong justification for our initial hypothesis that relevance in dynamics results in relevance of extracted information.

**Table 3:** Comparative Assessment with Other Clustering Methods

	*k-means*	*HC*	*STEM*	*Motifs*
% enriched GO	0.11	0.13	0.35	0.30
% inflammation-specific enriched GO	0.40	0.40	0.50	**0.79**
% enriched TFs	0.08	0.13	0.28	0.17
% inflammation-specific enriched TFs	0.25	0.28	0.28	**0.57**

The motifs, key regulatory elements, and ontologies we have identified may serve as a valuable basis for the identification of therapeutic options to detect as well as manage the onset of acute inflammation. Given the progress today in the areas of metabolic engineering and gene silencing, the therapeutic utilization of these genes can occur within two broad categories: 1) metabolically supplementing the patient suffering from acute inflammation, following burn injury in order to maintain the energetic levels of the liver required to produce acute phase proteins; 2) utilizing silencing techniques in order to control key regulatory elements we have identified, in order to mitigate the effects of acute inflammatory response that arises.

## Conclusion

We have presented a novel approach combining a fine-grained clustering and informative expression motifs identification. The key novelty of our methodology is the introduction of the concept of transcriptional state which allows the quantification of the deviations from a control state. Hence, we are able to measure the ability of expression motifs to capture deviations from the control state and, therefore, identify relevant components of the transcriptional response. The method was applied to the analysis of burn-induced inflammation based on a rat animal model. Our approach for informative expression profiles selection has identified motifs which characterize the inflammatory response as observed in liver during the first 24 hours after thermal injury. Significant processes identified, and associated with informative genes, involved in glycogen metabolism, catecholamine metabolism, ubiquitin dependent protein catabolism, as well as genes involved in the production of thioredoxin and N-acetylglucosamine. In addition, we identify critical regulators controlling the expression of the informative genes and we quantified the reconstructed activities of the corresponding transcription factors. We have demonstrated that our proposed methodology can significantly reduced the number of relevant probes while maintaining a high level of specificity in the processes that are identified.

## Methods

### Experimental Data

Experimental DNA microarray data is available at the Gene Expression Omnibus (GEO) database under the accession number GSE802. In this previously published study, male Sprague-Dawley rats were subjected to a cutaneous 3rd degree burn injury consisting of a full skin thickness scald burn of the dorsum, calculated to be ~20% of the rat's total body surface area [4]. Liver samples were obtained at 5 time points (0, 1, 4, 8, and 24 h post burn). RNA extracted from the extracted livers was isolated and subsequently hybridized to a Affymetrix U34A GeneChip that had 8,799 probes represented on each chip. The control for this experiment is the measurement labeled "Time 0" which was obtained prior to the thermal injury. It has been previously shown that time had no significant effect upon the response of rats to the sham treatment [8].

### Gene Expression Analysis

A comprehensive review of computational methods for the analysis of time series expression data was presented in [58,59]. These methods can be classified in two major families. Methods that measure the "distance" between members of different groups and "model-based" methods which assume the existence of an underlying model that describes the temporal dependencies in the data [59]. In [60] a novel algorithm extending the concept of time correlation to account also for time lagged and inverted relations among expressed genes was presented. In [61] the expression dynamics is modeled via autoregressive equations and agglomerative clustering procedures are used to search for the most probable set of clusters in the available data. The approach explores Bayesian concepts to account for the possible temporal dependencies of the expression data. In [62] a pattern recognition-based approach is used to capture similarity by finding salient changes in time-varying expression patterns of genes. It was proposed that such changes can give clues about important events such as regulation, cell cycle or disease onset. By and large, temporal expression profiling analysis is driven by the concept of similarity and focuses on aggregating expression profiles according to some metric quantifying the relative topologically similarity, correlation, or anti-correlation, of the features [55]. A general concern regarding the validity of existing algorithms stems from the practical observation that classification algorithms can lead to dubious results which are often method dependent [63]. Temporal transcription profiling is primarily aimed at identifying characteristics shared by genes exhibiting common dynamic responses [60,64]. For deciphering cell states and disease progression, only recently have researchers begun looking at the dynamics of gene ensembles and converging trajectories as high-dimensional attractors [37,65-67]. In order to thoroughly assess the progression of a disease and reveal the molecular events driving transcription changes representative of an organisms' response to external stimuli (i.e. burn), it is important to consider the ensemble of changes affecting the state of the organism as opposed to simply identifying components with similar temporal response. It does not simply suffice to consider the evolution of a particular gene expression over time, but rather we should consider the evolution of the entire state of the system over time. Current clustering algorithms aim at aggregating indiscriminately all available responses, whether relevant or not, instead of selecting among the available profiles, those that appear to be maximally affected by the specific perturbation. As a result, expression analysis in the field of burn induced inflammation has been primarily either descriptive, i.e. assembling all possible responses, or hypothesis-driven, i.e. specific targets are analyzed and verified [1,7,17]. We will propose a novel approach which combines the clustering of expression motifs and the selection of relevant responses in order to improve the information content of transcription analysis.

By combining classification and selection into one integrated step, we implicitly suggest that there is sufficient information about the relevance of a gene based solely upon its shape. While undoubtedly there is important information about a gene's relevance based upon the magnitude of its expression profile, it is not being explicitly considered in our algorithm. This was done in order to assess the informativeness of shape independent of other factors. This however does not preclude pre-processing of the input or post-processing of the results to take magnitude into account.

### Identification of major expression patterns

The expression data is given as an NxT matrix, E, where N is the number of probes and T is the vector of time points at which mRNA levels has been measured. For our analysis, we wish to characterize the entire expression waveform for each gene in the array. Therefore, we would like to assign to each waveform a characteristic attribute so that similarly shaped waveforms share similar attribute values. In many respects, this is reminiscent of the classical problem of analyzing multi-dimensional time series of which numerous approaches have been proposed in the literature and an extensive review is presented in [68]. We have adopted the basic formalism of **S**ymbolic **A**ggregate appro**X**imation of the time series discussed in [69] albeit with some modifications. **SAX **is based on the premise of transforming a time series into a corresponding sequence of symbols. Each series is first normalized as the z-score given as

Yj,t=Ej,t−μXjσXj;j=1,...,N;t=1,...,T     (1)
 MathType@MTEF@5@5@+=feaafiart1ev1aaatCvAUfKttLearuWrP9MDH5MBPbIqV92AaeXatLxBI9gBaebbnrfifHhDYfgasaacH8akY=wiFfYdH8Gipec8Eeeu0xXdbba9frFj0=OqFfea0dXdd9vqai=hGuQ8kuc9pgc9s8qqaq=dirpe0xb9q8qiLsFr0=vr0=vr0dc8meaabaqaciaacaGaaeqabaqabeGadaaakeaacqWGzbqwdaWgaaWcbaGaemOAaOMaeiilaWIaemiDaqhabeaakiabg2da9maalaaabaGaemyrau0aaSbaaSqaaiabdQgaQjabcYcaSiabdsha0bqabaGccqGHsisliiGacqWF8oqBdaWgaaWcbaGaemiwaG1aaSbaaWqaaiabdQgaQbqabaaaleqaaaGcbaGae83Wdm3aaSbaaSqaaiabdIfaynaaBaaameaacqWGQbGAaeqaaaWcbeaaaaGccqGG7aWocqWGQbGAcqGH9aqpcqaIXaqmcqGGSaalcqGGUaGlcqGGUaGlcqGGUaGlcqGGSaalcqWGobGtcqGG7aWocqWG0baDcqGH9aqpcqaIXaqmcqGGSaalcqGGUaGlcqGGUaGlcqGGUaGlcqGGSaalcqWGubavcaWLjaGaaCzcamaabmaabaGaeGymaedacaGLOaGaayzkaaaaaa@59E9@

An equiprobable discretization technique is then applied where the breakpoints are defined such that the area defined by the boundaries of the breakpoint and the Gaussian curves are equal. Breakpoints that divide the Gaussian distribution into regions of equal area are obtained through statistical tables which give the values of the cumulative distribution function (CDF) of the N(0,1) distribution. The CDF values can also be calculated via a standard formula by solving for b:

ik−1=12[1+erf(b2)];     (2)
 MathType@MTEF@5@5@+=feaafiart1ev1aaatCvAUfKttLearuWrP9MDH5MBPbIqV92AaeXatLxBI9gBaebbnrfifHhDYfgasaacH8akY=wiFfYdH8Gipec8Eeeu0xXdbba9frFj0=OqFfea0dXdd9vqai=hGuQ8kuc9pgc9s8qqaq=dirpe0xb9q8qiLsFr0=vr0=vr0dc8meaabaqaciaacaGaaeqabaqabeGadaaakeaadaWcaaqaaiabdMgaPbqaaiabdUgaRjabgkHiTiabigdaXaaacqGH9aqpdaWcaaqaaiabigdaXaqaaiabikdaYaaadaWadaqaaiabigdaXiabgUcaRiabdwgaLjabdkhaYjabdAgaMnaabmaabaWaaSaaaeaacqWGIbGyaeaadaGcaaqaaiabikdaYaWcbeaaaaaakiaawIcacaGLPaaaaiaawUfacaGLDbaacqGG7aWocaWLjaGaaCzcamaabmaabaGaeGOmaidacaGLOaGaayzkaaaaaa@44DE@

*i *= 1,..,*k*; *k *= number of breakpoints; b = breakpoint value

**Figure 7:**
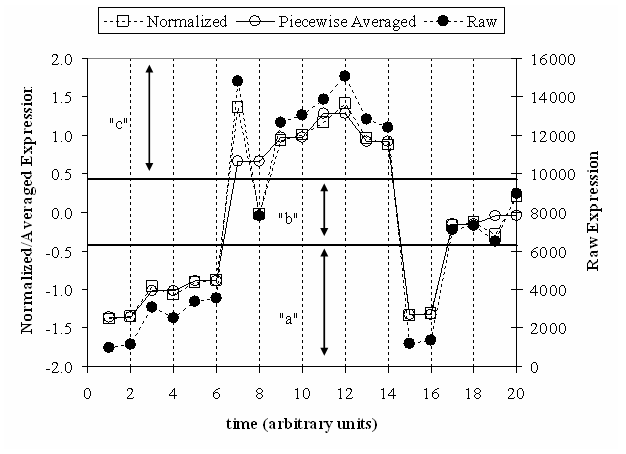
An example of a HOT-SAX transformation of a time series (w = 2, α = 3).

This method of discretization was selected because empirical evidence suggests that the z-score normalized sub-patterns should have a highly Gaussian distribution [69], thereby equally distributing a set of randomly generated signals throughout the hash space. Coefficients below the smallest breakpoint are "mapped" to the first symbol of a chosen alphabet (for example a). Other points are "mapped" accordingly within their respective intervals. A more extensive discussion and visualization of this process and can be found in [69]. The elements of the symbolic transformation are exemplified in Figure 7. This symbolic representation makes it possible to further simplify the time series in order to uniquely characterize the overall dynamic response of each transcriptional profile as a single number through hashing [70]. After the alphabet has been generated, it is condensed into a single hash value using the function proposed by [71]:

hash(c,w,a)=1+∑j=1w[ord(cj)−1]×aj−1     (3)
 MathType@MTEF@5@5@+=feaafiart1ev1aaatCvAUfKttLearuWrP9MDH5MBPbIqV92AaeXatLxBI9gBaebbnrfifHhDYfgasaacH8akY=wiFfYdH8Gipec8Eeeu0xXdbba9frFj0=OqFfea0dXdd9vqai=hGuQ8kuc9pgc9s8qqaq=dirpe0xb9q8qiLsFr0=vr0=vr0dc8meaabaqaciaacaGaaeqabaqabeGadaaakeaacqWGObaAcqWGHbqycqWGZbWCcqWGObaAcqGGOaakcqWGJbWycqGGSaalcqWG3bWDcqGGSaalcqWGHbqycqGGPaqkcqGH9aqpcqaIXaqmcqGHRaWkdaaeWbqaaiabcUfaBjabd+gaVjabdkhaYjabdsgaKjabcIcaOiabdogaJnaaBaaaleaacqWGQbGAaeqaaOGaeiykaKIaeyOeI0IaeGymaeJaeiyxa0Laey41aqRaemyyae2aaWbaaSqabeaacqWGQbGAcqGHsislcqaIXaqmaaaabaGaemOAaOMaeyypa0JaeGymaedabaGaem4DaChaniabggHiLdGccaWLjaGaaCzcamaabmaabaGaeG4mamdacaGLOaGaayzkaaaaaa@5B1E@

where *a *is the size of the alphabet, *w *is length of the word, and *c *is the "letter" sequence to which the expression profile is assigned. This is essentially the conversion of a base *a *number into base 10 with a change of making the smallest value 1 instead of 0. The only difference between our hashing method and the originally proposed method is the change in the most significant bit (MSB). By having the MSB as the first letter in our word, rather than having the most significant bit as the last letter in our word, we weigh the differences of the sequences at the beginning heavier than that of the end. Consistent with this is the observation that the signals that were correlated at the beginning of the time points were more closely related than signals that were more correlated at the end of the time series. In our analysis we experimented with various combinations of the two major parameters required, *w *and *a*. The results presented are based on an alphabet, *a*, size of 4, and the word size, *w*, of 5. Given that we are attempting to find non-random expression profiles within a set of gene expression profiles, the distribution of hash values must be non-random, i.e. non-exponential. Therefore for the selection of alpha, we would generate all of the hash values, and then determine the distribution of the populations of hash values and then evaluate how well these distributions can be fitted by an exponential distribution, and select the alpha which gives the worst fit (most non-exponential) which in our case is at α = 4.

What has been achieved at this point is the assignment of a unique identifier to all the transcriptional profiles. Therefore, genes with similar normalized expression profiles "hash" to similar motif values. As a result, we can generate a distribution of such motif values and identify (i) dominant, i.e., overpopulated motifs, and (ii) genes sharing similar motif values, i.e., sharing similar expression profiles. Hence we have achieved a very fine-grained "clustering" of the data where the number of potential clusters is dependent upon the definition of the hashing function.

### Characterization the transcriptional state of the system and extraction of the most informative expression patterns

Having assigned the expression profiles to distinct motifs, the next task is to identify the motifs that are maximally affected by the experimental perturbation. We first define a concept we term the "transcriptional state", which is the statistical distribution of the expression levels at a specific time point. The motivation for using this concept is that the genes which react to a stimulus will be either strongly up or down regulated. Therefore, there should be a significant change in the distribution of expression levels in a set of informative genes compared to the distribution of expression levels of uninformative genes. Had we considered the totality of the transcriptional information, it would have been rather clear that the expression intensities of all the probes, averaged between replicate arrays, and plotted over time would have been practically the same for all time points, as [4] point out for the system under study (we specifically refer to Figure 1 in [4]). Therefore, even though gene expression measurements do contain information, this is confined to only a sub-set of genes which we have to identify in a rigorous and systematic way.

To quantify the hypothesis that informative subsets of genes should give rise to distribution of expression values maximally affected by the experiment, the Kolmogorov-Smirnov (K-S) test which is a standard test for evaluating whether or not two distributions are different, is employed. The K-S test is applicable to un-binned, arbitrary and unknown distributions that are functions of a single independent variable (that is, each data point can be associated with a single number). The list of data points (the ensemble of the expression values of all the genes at each time point) can be easily converted to an unbiased estimator of the cumulative distribution function of the expression levels from which the data was drawn. The fundamental concept is that truly informative subsets of genes are the ones that have the ability to capture significant deviations from the base distribution.

The K-S test was selected over other statistical tests which are used in order to differentiate between statistical distributions due mainly to its ability to work on arbitrary distribution. Tests such as the Lilliefors test may improve upon the discriminant nature of the K-S test, but rely upon the use of known distributions. By utilizing the KS statistic, we make no assumption as to the underlying distribution of our data, and can therefore utilize a completely data dependent metric.

The K-S test is a very simple yet effective way of comparing two distributions and has found many widespread applications [72]. The K-S test quantifies a particularly simple measure: it is defined as the maximum absolute difference between two cumulative distribution functions. In our setting, and for each time point, we estimate a cumulative distribution function (CDF) of the expression values by appropriate binning of the expression values. The base distribution is the corresponding CDF prior to the injury. The K-S statistic is defined as:

D=max1≤i≤n|F(Yi)−F(Yi(0))|     (4)
 MathType@MTEF@5@5@+=feaafiart1ev1aaatCvAUfKttLearuWrP9MDH5MBPbIqV92AaeXatLxBI9gBaebbnrfifHhDYfgasaacH8akY=wiFfYdH8Gipec8Eeeu0xXdbba9frFj0=OqFfea0dXdd9vqai=hGuQ8kuc9pgc9s8qqaq=dirpe0xb9q8qiLsFr0=vr0=vr0dc8meaabaqaciaacaGaaeqabaqabeGadaaakeaacqWGebarcqGH9aqpdaWfqaqaaGqaciab=1gaTjab=fgaHjab=Hha4bWcbaGaeGymaeJaeyizImQaemyAaKMaeyizImQaemOBa4gabeaakmaaemaabaGaemOrayKaeiikaGIaemywaK1aaSbaaSqaaiabdMgaPbqabaGccqGGPaqkcqGHsislcqWGgbGrcqGGOaakcqWGzbqwdaWgaaWcbaGaemyAaKgabeaakiabcIcaOiabicdaWiabcMcaPiabcMcaPaGaay5bSlaawIa7aiaaxMaacaWLjaWaaeWaaeaacqaI0aanaiaawIcacaGLPaaaaaa@4FDE@

where F(Y_i_(0)) is the cumulative distribution of the expression values at time t = 0. This statistic allows a metric that defines the magnitude of the difference between two distributions to be computed. Since the data is presented as a time series, at each time point a value for the Kolmogorov statistic is obtained. To condense the N values into a single numeric score, we utilize the infinity norm. Therefore, the overall metric then becomes

D=max⁡tmax⁡1≤i≤n|F[Yi(t)]−F[Yi(0)]|     (5)
 MathType@MTEF@5@5@+=feaafiart1ev1aaatCvAUfKttLearuWrP9MDH5MBPbIqV92AaeXatLxBI9gBaebbnrfifHhDYfgasaacH8akY=wiFfYdH8Gipec8Eeeu0xXdbba9frFj0=OqFfea0dXdd9vqai=hGuQ8kuc9pgc9s8qqaq=dirpe0xb9q8qiLsFr0=vr0=vr0dc8meaabaqaciaacaGaaeqabaqabeGadaaakeaacqWGebarcqGH9aqpdaWfqaqaaiGbc2gaTjabcggaHjabcIha4bWcbaGaemiDaqhabeaakmaaxababaGagiyBa0MaeiyyaeMaeiiEaGhaleaacqaIXaqmcqGHKjYOcqWGPbqAcqGHKjYOcqWGUbGBaeqaaOWaaqWaaeaacqWGgbGrcqGGBbWwcqWGzbqwdaWgaaWcbaGaemyAaKgabeaakiabcIcaOiabdsha0jabcMcaPiabc2faDjabgkHiTiabdAeagjabcUfaBjabdMfaznaaBaaaleaacqWGPbqAaeqaaOGaeiikaGIaeGimaaJaeiykaKIaeiyxa0facaGLhWUaayjcSdGaaCzcaiaaxMaadaqadaqaaiabiwda1aGaayjkaiaawMcaaaaa@5A79@

The application of the K-S test over time allows us to quantify just how much the CDF of a particular sub-set of genes deviates from the corresponding CDF at time t = 0 (control). The most sensitive sub-set exhibits the largest deviations from the control. Once the subset is specified then it can be characterized based on its corresponding D value. We have currently implemented a simple greedy algorithm that selects peaks based on their population. The basic steps of the algorithm are as follows:

(i) *k *= 0, *S*(*k*) = ∅, *D*(*k*) = -∞ max = -∞

(ii) *k *= *k *+ 1

(iii) h*, arg max *N*(*h*), *N*(*h*) = number of genes with corresponding hash value *h*

(iv) *G*(*k*) = {*g*_*i*_: *hash*(*g*_*i*_) = h*}, the subset of genes that hash to *h*

(v) Evaluate *F*(*Y*_*gi*_(*t*)); *t *= 0,...,*T*; *g*_*i *_∈ Σ

(vi) Evaluate D(k)=max⁡tmax⁡gi∈Σ|F[Ygi(t)]−F[Ygi(0)]|
 MathType@MTEF@5@5@+=feaafiart1ev1aaatCvAUfKttLearuWrP9MDH5MBPbIqV92AaeXatLxBI9gBaebbnrfifHhDYfgasaacH8akY=wiFfYdH8Gipec8Eeeu0xXdbba9frFj0=OqFfea0dXdd9vqai=hGuQ8kuc9pgc9s8qqaq=dirpe0xb9q8qiLsFr0=vr0=vr0dc8meaabaqaciaacaGaaeqabaqabeGadaaakeaacqWGebarcqGGOaakcqWGRbWAcqGGPaqkcqGH9aqpdaWfqaqaaiGbc2gaTjabcggaHjabcIha4bWcbaGaemiDaqhabeaakmaaxababaGagiyBa0MaeiyyaeMaeiiEaGhaleaacqWGNbWzdaWgaaadbaGaemyAaKgabeaaliabgIGiolabfo6atbqabaGcdaabdaqaaiabdAeagjabcUfaBjabdMfaznaaBaaaleaacqWGNbWzdaWgaaadbaGaemyAaKgabeaaaSqabaGccqGGOaakcqWG0baDcqGGPaqkcqGGDbqxcqGHsislcqWGgbGrcqGGBbWwcqWGzbqwdaWgaaWcbaGaem4zaC2aaSbaaWqaaiabdMgaPbqabaaaleqaaOGaeiikaGIaeGimaaJaeiykaKIaeiyxa0facaGLhWUaayjcSdaaaa@5BBB@

(vii) If D(k) > max

(viii) Max = D(k); F = k;

(ix) Go to (ii) until all peaks have been added

(x) For a = 1 to F

(xi) Select Σ = *S*(*a *- 1)∪*G*(*a*)

The iteration count *k *corresponds to the number of peaks that are incorporated at each step. *S(k) *is the set of hash values that have been considered up to iteration *k*. *N(h) *is the number of genes that have been assigned to a particular hash value *h*, h* is the motif values that is most populated at each iteration. *G(k) *is the subset of genes, *g*_*i*_, that have hashed to h, while S is the cumulative set of genes included at each iteration. *D(k) *is the K-S statistic evaluated at iteration k and is calculated using the set *S *of genes. Once a peak and its corresponding genes, has been included then the corresponding hash value is eliminated so that it is not considered again in the future. The search is performed upon motifs which are comprised up of genes with similar expression profiles, as opposed to individual genes. The peaks (along with the corresponding genes) are added provided that a clear deviation from the control state is observed.

The two elements just described (identification of major expression patterns and characterization of the transcriptional state) define the elements of a novel fine-grained selection/clustering algorithm which permits the identification of groups of genes whose expression motifs are maximally affecting the underlying dynamic of the transcriptional experiment as defined by the CDF of the corresponding expression patterns of the selected genes.

### Randomized Testing

To validate the fact that the proposed algorithm, especially the use of the KS statistic is selecting meaningful dynamics, it was important to evaluate the behavior of the KS statistic over randomly selected motifs. To prove that the statistic was not driven primarily by the number of genes selected, motifs were randomly selected until the set of genes was the same as the number of informative genes, after which the KS Statistic would be evaluated. Additionally to verify that the selection of motifs is a reasonable approach, we then selected random genes corresponding to the same number of informative genes and evaluate the KS statistic. A positive result in both cases would show a maximum KS statistic below that the informative result.

### Functional Characterization of Informative Motifs

To validate the biological foundations for the results, we utilized ontology enrichment analysis. Given the fact that our algorithm is performing a selection and grouping of informative genes, statistically over-represented ontologies ought to provide a list of important underlying phenomenon which is part of the mechanism behind the organism's response to burn. This is done in order to complement the results obtained from mining the Kyoto Encyclopedia of Genes and Genomes (KEGG) database. Given the incomplete identification of pathways, we felt than an overall summary of important processes would be helpful in providing a better view as to the necessary compensatory mechanisms.

The gene ontology formulism which we have selected is based upon the hypergeometric distribution [73]. The hypergeometric distribution is based upon the binomial distribution and calculate the probability that a given number of ontologies will localized to a given cluster given the total number of times the ontology is present within the cluster, the number of times an ontology is present within the entire dataset, the number of genes in the cluster of interest, and the total number of genes. For ontologies in which the expected number of occurrences was less than 5, we used the 1-tailed Fisher test as stated by[73]. The equation for the hypergeometric distribution is given in Equation 6, and the equation for the Fisher test is given in Equation 7.

P=1−∑k=1n(ki)(m−kN−i)(mN);     (6)
 MathType@MTEF@5@5@+=feaafiart1ev1aaatCvAUfKttLearuWrP9MDH5MBPbIqV92AaeXatLxBI9gBaebbnrfifHhDYfgasaacH8akY=wiFfYdH8Gipec8Eeeu0xXdbba9frFj0=OqFfea0dXdd9vqai=hGuQ8kuc9pgc9s8qqaq=dirpe0xb9q8qiLsFr0=vr0=vr0dc8meaabaqaciaacaGaaeqabaqabeGadaaakeaacqWGqbaucqGH9aqpcqaIXaqmcqGHsisldaaeWbqaamaalaaabaWaaeWaaeaafaqabeGabaaabaGaem4AaSgabaGaemyAaKgaaaGaayjkaiaawMcaamaabmaabaqbaeqabiqaaaqaaiabd2gaTjabgkHiTiabdUgaRbqaaiabd6eaojabgkHiTiabdMgaPbaaaiaawIcacaGLPaaaaeaadaqadaqaauaabeqaceaaaeaacqWGTbqBaeaacqWGobGtaaaacaGLOaGaayzkaaaaaaWcbaGaem4AaSMaeyypa0JaeGymaedabaGaemOBa4ganiabggHiLdGccqGG7aWocaWLjaGaaCzcamaabmaabaGaeGOnaydacaGLOaGaayzkaaaaaa@4DB3@

n = number of times the ontology appears in a given cluster

i = number of genes in a given cluster

N = total number of genes

m = number of times the ontology appears in the dataset

P=∑c=1n11R1!R2!C1!C2!N!c!(n12−c)!n21!n22!     (7)
 MathType@MTEF@5@5@+=feaafiart1ev1aaatCvAUfKttLearuWrP9MDH5MBPbIqV92AaeXatLxBI9gBaebbnrfifHhDYfgasaacH8akY=wiFfYdH8Gipec8Eeeu0xXdbba9frFj0=OqFfea0dXdd9vqai=hGuQ8kuc9pgc9s8qqaq=dirpe0xb9q8qiLsFr0=vr0=vr0dc8meaabaqaciaacaGaaeqabaqabeGadaaakeaacqWGqbaucqGH9aqpdaaeWbqaamaalaaabaGaemOuai1aaSbaaSqaaiabigdaXaqabaGccqGGHaqicqWGsbGudaWgaaWcbaGaeGOmaidabeaakiabcgcaHiabdoeadnaaBaaaleaacqaIXaqmaeqaaOGaeiyiaeIaem4qam0aaSbaaSqaaiabikdaYaqabaGccqGGHaqiaeaacqWGobGtcqGGHaqicqWGJbWycqGGHaqicqGGOaakcqWGUbGBdaWgaaWcbaGaeGymaeJaeGOmaidabeaakiabgkHiTiabdogaJjabcMcaPiabcgcaHiabd6gaUnaaBaaaleaacqaIYaGmcqaIXaqmaeqaaOGaeiyiaeIaemOBa42aaSbaaSqaaiabikdaYiabikdaYaqabaGccqGGHaqiaaaaleaacqWGJbWycqGH9aqpcqaIXaqmaeaacqWGUbGBdaWgaaadbaGaeGymaeJaeGymaedabeaaa0GaeyyeIuoakiaaxMaacaWLjaWaaeWaaeaacqaI3aWnaiaawIcacaGLPaaaaaa@5CC0@

R_1 _= Number total amount of ontologies present

R_2 _= Number of the ontology of Interest

C_1 _= Number of Genes in the Raw Dataset

C_2 _= Number of Genes in the Cluster

N = R_1 _+ R_2 _= C_1 _+ C_2_

*n*_11 _= Number of genes in the cluster with the ontology

*n*_12 _= Number of genes in the cluster

*n*_21 _= Number of genes in the raw data with the ontology

*n*_22 _= Number of genes in the raw data without the ontology

After the probability of each ontology present is calculated, we then take the ontologies which are most statistically significant (p < .05) and perform further analysis upon these ontologies in order to identify significant processes which take place. A similar analysis was conducted for transcription factors in order to determine whether or not transcription factors were preferentially localized to a specific cluster. The initial list of transcription factors were extracted via trafac [30] with a promoter region of 200 base pairs upstream of the start codon. We selected 200 base pairs upstream for our transcription factor analysis given the results from [74], that suggested that the region of maximum promoter sequence homology between rats and mice was at 200 base pairs or less upstream of the start codon.

In order to visualize the distribution of ontologies and transcription factors, we have constructed an image where significant ontologies are coded in green and ontologies which are not statistically significant are coded in black. As a post processing step, we sort the matrix in the same fashion that a radix sort works, i.e. first sorting by the last column, then iterative moving to the next to last column, until the first column is reached[70]. This will then arrange our ontologies in such a manner where the significant ontologies for each cluster will be grouped together. Therefore, if a significant number of ontologies are localized to each cluster, then we should obtain a diagonally dominant plot.

### Building Gene Interaction Networks

Genes belonging to informative motifs were subsequently fed into Pathway Assist in order to assess their functional relations. Pathway Assist [75] is a software application developed for navigation and analysis of biological pathways, gene regulation networks, and protein interaction maps. It comes with the built-in natural language processing module MedScan and a comprehensive database describing more than 100,000 events of regulation, interaction and modification between proteins, cell processes, and small molecules. Pathway Assist mines papers indexed on PubMed for gene names, and genes that have been mentioned in the same paper are assumed to be related and therefore a connection can be drawn between the two genes. As a result, a plausible network of interactions is created. Protein interaction networks, for each of the 6 informative clusters, were built using Pathway Assist and further analyzed using the basic functionalities of Cytoscape. The interactions were established so that complete paths were established between all genes. The networks of interactions are highly complex and visual inspection is uninformative. However, further analysis of the interaction maps reveals some important properties. The inherent structure of the graphs was determined by evaluating the degree distribution. The distribution for all clusters clearly follows as power-law (P(k) = αk^-γ ^with values for the degree exponent γ indicative of a scale-free network. The value of the degree exponent is a critical characteristic determining important properties of the graph. The smaller the value of g the more important the role of the hubs (nodes with high connectivity) is in the network [76]. The degree values we identify are well bellow the threshold for scale-free networks of 3 [77] indicating the existence of such highly connected nodes.

### Elucidation and Quantification of Regulatory Interactions

After the identification of possible links through transcription factor analysis, we need to carry out the quantification of these links. The identification of these links through transcription factor analysis only provides a superset of possible interactions which are occurring in the experimental system. However, after the quantification of these links we can begin to assign the significance of each link in order to obtain a reduced set of connections that are active within the experimental regime. This allows for the identification of significant pathways which function as the primary driving forces of the system. This information can perhaps lead to possible points of control in order to mitigate the detrimental responses of an organism. However, physical binding of a TF is a necessary but not sufficient condition for transcription initiation and regulation. Due to various complex post-translational modifications as well as interactions among multiple TFs the measured expression level of regulatory genes does not reflect the actual activity of the TFs themselves. Therefore, regulator transcription levels are generally not appropriate measures of transcription factor activity (TFA). Recently, methods combining TF-gene connectivity data and gene expression measurements have emerged in order to quantify these regulatory interactions. Prominent examples are the decomposition-based methods which combine ChiP and microarray data and inversion of regression techniques to estimate TFAs [78-81]

Numerous statistical techniques have been proposed recently for the construction of lower-dimensional representation regulatory networks from high-dimensional gene expression data [82,83]. Network Component Analysis (NCA) [84,85] was a recently proposed method for the quantification of regulatory interactions and the estimation of transcription factor activities. It offers the advantage over more commonly used regulatory model building techniques in that it does not rely upon purely mathematical assumptions that other decomposition methods do. In the commonly used component analyses such as principal component analysis (PCA) and independent component analysis (ICA), assumptions are made on the matrix of basis vectors such as orthogonality or statistical independence. These assumptions are usually not borne out by the underlying biology. NCA on the other hand takes its assumption from the underlying biological structure, namely predicted transcription factor binding sites, which makes it much better suited for quantifying networks in a relevant manner.

The formulation of NCA is as follows: Given a set of log normalized temporal expression profiles [E](NxT), there exists a decomposition [A](NxL) and [P](LxT) where [A] is the connectivity matrix and [P] is the basis matrix representing transcription factor activity, N is the number of genes, T the number of time points and L the number of transcription factors. We obtain the overall connectivity matrix by processing the results obtained via TRAFAC. The regulatory weights are zero if TF_j _does not regulate gene *i*, and a non-zero value if TF_j _regulates gene i. Therefore NCA imposes structure to matrix [A] derived from transcription factor analysis, giving it a biological rather than a mathematical basis to for the decomposition [85]. This is in contrast to the other commonly used decomposition methods such as PCA or ICA. In PCA, the set of components are assumed to be orthogonal, and in ICA the set of components are assumed to be statistically independent [86]. In NCA, there is no assumption about the structure of [P]. Rather the assumption has been moved to [A], which we are able to extract information via transcription factor analysis. In order to satisfy the goal that the entire solution space of NCA will differ by only a diagonal scaling matrix, the following additional constraints must be satisfied: (i) [A] must be of full column rank; (ii) the reduced form of [A] must also be of full column rank; and (iii) [P] must be of full rank. The interested reader should consult the original presentation of NCA [84,85] for a thorough discussion of the aforementioned constraints and their implications.

Given the density of the initial transcription factor binding matrix, obtained through transcription factor analysis, it is unlikely that the initial matrix will be NCA compliant, thus satisfying the three aforementioned constraints. [84,85] suggest an iterative process in which connections are randomly deleted from the initial connectivity matrix and checked to see if the resultant matrix is NCA compliant[87].

### Relation to Previous Work

A preliminary exposition of the basic elements of the approach was presented in [88]. The current publication significantly expands the previous work in a number of ways.

1. In [88] an *ad hoc *analysis was presented in an attempt to verify the presence of genes which were known to play some role in thermal injury and inflammation. A rudimentary analysis of over-represented gene ontologies and transcription factors, and not a rigorous enrichment analysis, was discussed to simply evaluate the potential of the analysis method. In the present work we performed a thorough enrichment analysis in order to identify relevant over-represented processes within the selected genes, as well as significant transcription factors through variance analysis of predicted transcription factor activity. Of particular importance are the results of Tables 1 and 2 that quantify the p-values of the corresponding enrichment.

2. In [88] we selectively verified the presence of a very limited number of already known relevant processes and discussed their implications. In the present work a thorough analysis of numerous processes and predicted transcription factors was performed, their potential role in the inflammatory process was discussed and an integrated picture was hypothesized.

3. Of particular importance are the transcription factor activity reconstruction data and most notably the results of Figure 5. In [88] we simply comment on the potential of NCA (Network Component Analysis) to reconstruct gene expression profiles. However, in the current manuscript we demonstrate how TFA profiles can be reconstructed.

4. New and very significant is the analysis summarized in Table 3, which compares our results to known clustering methods. A number of different approaches were implemented, including recent methods specifically developed for temporal gene expression data and methods were evaluated based on their ability to enrich GO and TFs in general, as well as their ability in enriching inflammation-specific GO and TFs. The results clearly indicate the superiority of our approach.

The improvements in the analysis steps are neither trivial nor auxiliary. In our previous work [88] we merely sought to verify the fact that our results were not nonsensical. While the data analyzed in both papers is the same, we wanted to create an automated method in which one could go from temporal expression data to a set of testable hypotheses with minimum human intervention. Therefore the methods which we proposed for the analysis are extremely important. The greater in depth analysis presented in our paper reflects this. Due to the automated analysis tools, we were able to obtain a greater understanding of the underlying response than before. With the current framework in both the extraction of informative genes, dominant motifs, and computational analysis of the results we are confident in our ability to discern major pathways, processes, and transcriptional signals that drive an unknown system. Therefore, in [88] we have not been able to synthesize any new knowledge about the system. However, in the present work, we believe we have been able to synthesize new information about an organism's response, namely the primary factors that drive the system, the time course in which these factors are active, and a time frame in which the system undergoes state changes in which treatment protocols would need to change due to differences in the transcriptional state. These are things which were not identified in the first iteration, nor could have been identified given the analysis framework in place at that time.

## List of Abbreviations

HIF – Hypoxia Inducible Factor

TNF – Tumor Necrosis Factor

IL-1 – Interleukin 1

TF – Transcription Factor

CIZ – Cas interacting Zinc Finger Protein

BMP – Bone Morphogenic Protein

AhR – Aryl Hydrocarbon Receptor

STEM – Short Time Expression Miner

SAX – Symbolic Aggregate Approximation

MSB – Most Significant Bit

KS – Kolmogorov-Smirnov

KEGG – Kyoto Encyclopedia of Genes and Genomes

NCA – Network Component Analysis

PCA – Principal Component Analysis

ICA – Independent Component Analysis

## Authors' contributions

IPA organized the study, EY conducted all computations and analysis, TM and FB provided the inflammation-related interpretations, MLY and FB provided the experimental data. All authors have read and approved the manuscript.
